# Abnormal degree centrality in first-episode medication-free adolescent depression at rest: A functional magnetic resonance imaging study and support vector machine analysis

**DOI:** 10.3389/fpsyt.2022.926292

**Published:** 2022-09-29

**Authors:** Xin Guo, Wei Wang, Lijun Kang, Chang Shu, Hanpin Bai, Ning Tu, Lihong Bu, Yujun Gao, Gaohua Wang, Zhongchun Liu

**Affiliations:** ^1^Department of Psychiatry, Renmin Hospital of Wuhan University, Wuhan, Hubei, China; ^2^Department of Psychosis Studies, Institute of Psychiatry, Psychology and Neuroscience, King’s College of London, London, United Kingdom; ^3^PET/CT/MRI and Molecular Imaging Center, Renmin Hospital of Wuhan University, Wuhan, Hubei, China

**Keywords:** adolescent depression, resting state, functional magenetic resonance imaging, degree centrality, support vector machine (SVM)

## Abstract

**Background:**

Depression in adolescents is more heterogeneous and less often diagnosed than depression in adults. At present, reliable approaches to differentiating between adolescents who are and are not affected by depression are lacking. This study was designed to assess voxel-level whole-brain functional connectivity changes associated with adolescent depression in an effort to define an imaging-based biomarker associated with this condition.

**Materials and methods:**

In total, 71 adolescents affected by major depressive disorder (MDD) and 71 age-, sex-, and education level-matched healthy controls were subjected to resting-state functional magnetic resonance imaging (rs-fMRI) based analyses of brain voxel-wise degree centrality (DC), with a support vector machine (SVM) being used for pattern classification analyses.

**Results:**

DC patterns derived from 16-min rs-fMRI analyses were able to effectively differentiate between adolescent MDD patients and healthy controls with 95.1% accuracy (136/143), and with respective sensitivity and specificity values of 92.1% (70/76) and 98.5% (66/67) based upon DC abnormalities detected in the right cerebellum. Specifically, increased DC was evident in the bilateral insula and left lingual area of MDD patients, together with reductions in the DC values in the right cerebellum and bilateral superior parietal lobe. DC values were not significantly correlated with disease severity or duration in these patients following correction for multiple comparisons.

**Conclusion:**

These results suggest that whole-brain network centrality abnormalities may be present in many brain regions in adolescent depression patients. Accordingly, these DC maps may hold value as candidate neuroimaging biomarkers capable of differentiating between adolescents who are and are not affected by MDD, although further validation of these results will be critical.

## Introduction

Major depressive disorder (MDD) is a highly prevalent yet deleterious psychiatric illness that can impair the psychological and social functioning of affected patients, reducing the overall quality of life and imposing a major burden on individuals suffering from this disease (1). First-episode MDD most often manifests between the middle of adolescents and the mid-40s, with an estimated 40% of first episodes occurring before the age of 20 (2). According to one recent meta-analysis, approximately 17.2% of children and adolescents between the ages of 6 and 15 in China have reported symptoms consistent with depression (3). Depression that occurs during adolescence, which is defined as 10–19 years of age by the World Health Organization (WHO), is more likely to be overlooked than depression in adults such that publically available statistics fail to accurately reflect the true burden of this illness (4, 5). In addition to cultural concerns pertaining to stigma and loss of privacy (6), the low rates of detection for adolescent depression are also driven by its highly heterogeneous presentation, which can manifest in the form of reactive moods, aggressive behaviors, and irritability with comorbid reductions in academic performance, eating disorders, anxiety, and other behavioral issues (7). As such, precisely detecting and diagnosing depression remains very challenging in this age group. Patterned symptom identification is an intrinsic component of MDD diagnosis under the Diagnostic and Statistical Manual of Mental Disorders (DSM) and International Classification of Diseases (ICD) diagnostic symptoms, yet these symptoms are not reliably associated with adolescent depression, and are also observed in the context of a range of other physical and mental ailments (8). Moreover, while the diagnosis of patients based on clinical descriptions has been shown to exhibit validity, it is associated with relatively poor specificity, contributing to the potential for diagnostic uncertainty, which can make appropriate clinical decision-making more challenging. There is thus an urgent need to gain further insight into the mechanistic basis for adolescent depression and to define robust and reliable means of detecting this condition.

Several recent neuroimaging studies have shown that young adults with MDD exhibit a range of neural functional connectivity analyses as compared to healthy control (HC) individuals. In a systematic review of functional brain imaging studies focused on young MDD patients published recently, these patients were found to exhibit changes in emotional processing, affective cognition, cognitive control, reward processing, and resting-state functional connectivity. While relatively few differences were observed when comparing younger adolescents and older youths, a comparison of youths with adult MDD patients revealed significant differences in the affective cognition and cognitive control domains (9). A meta-analysis of functional neuroimaging findings from young MDD patients revealed abnormal activation in several executive functions and affective processing tasks relative to HC individuals (10). These results suggest that differences in brain connectivity may offer a more objective opportunity to diagnose adolescents suffering from MDD.

Degree centrality (DC) is an index that measures whole-brain connectivity based on a global description of the characteristics of a particular region of the grain through analyses of functional connectivity between that region and the brain as a whole based on graph theory measures. DC analyses have recently been employed as a means of defining the core architectural conformation of brain networks, with higher and lower levels of DC in particular brain areas corresponding to increased and decreased global connectivity, respectively (11). Here, voxel-wise DC values were used to explore neuroimaging abnormalities in adolescents affected by depression. This approach offers great promise given that DC has been successfully used to examine whole-brain changes associated with conditions such as MDD (12–14), schizophrenia (15, 16), autism spectrum disorder (ASD) (17), and bipolar disorder (BPD) (12, 18, 19), providing a highly sensitive, specific, and reproducible biomarker that is physiologically meaningful. While DC holds great promise as a means of comprehensively analyzing brain networks in MDD patients, studies defining adolescent depression-specific changes in DC are lacking. Accordingly, the main goal of this study was to define specific neuroimaging biomarkers of MDD in adolescents.

Machine learning techniques offer a key advantage when analyzing large-scale, complex datasets. Support vector machine (SVM) machine learning methods, which were developed using statistical learning theory, have been successfully used to aid in the diagnosis and prediction of therapeutic responses or prognostic outcomes (20) for conditions such as ASD (21), BPD (22, 23), MDD (12, 24), and schizophrenia (25–27) using both structural and functional neuroimaging data. SVM techniques are the most commonly employed machine learning methods in the context of brain imaging classification and depression given that they exhibit a robust theoretical foundation and can flexibly respond to high-dimensional data (28). SVM approaches are also ideally suited to small sample sizes and the recognition of non-linear, high-dimensional patterns (29).

Here, a kernel SVM was employed to detect voxel-wise DC changes when comparing 71 unmedicated adolescent first-episode MDD patients to 72 age-, gender-, and educational level-matched HCs, with the goal of developing a stronger understanding of the mechanisms governing MDD and providing a new approach to effectively diagnosing this condition.

## Materials and methods

### Participants

#### Ethical oversight

The Renmin Hospital of Wuhan University (Wuhan, China) ethics committee approved this study, which was consistent with the Declaration of Helsinki (Version 2002). All participants and their legal guardians provided written informed consent after receiving a full study description.

#### Recruitment and assessment

Patients participating in this study were recruited through the Center of Prevention and Management of Depression in Hubei Province, Renmin Hospital of Wuhan University through advertising and word-of-mouth interactions with past and current patients and volunteers. Data collection ran from May 4, 2018 through December 30, 2018. In total, 71 adolescents diagnosed with MDD as per the DSM-IV were recruited for this study, with diagnoses having been confirmed *via* the Structured Clinical Interview for DSM-IV Axis I disorders (SCID) by two board-certified psychiatrists. To be eligible for participation, patients had to meet the following criteria: (1) 14–18 years of age; (2) a history of MDD illness < 12 months; (3) no history of depression-related medication or electroconvulsive therapy; (4) no diagnoses of other mental health conditions as per the DSM-IV diagnostic criteria; (5) no instances of head trauma that resulted in the loss of consciousness; (6) no current or prior somatic illnesses with the potential to impact brain morphology; (7) no history of substance abuse; and (8) right-handed. Patients were excluded if: (1) they exhibited any serious physical or neurological conditions, somatic disease, brain morphological abnormalities, or a history of drug or alcohol abuse; (2) were ineligible for MRI due to the surgical placement of metal or electronic materials; (3) have utilized any medications within the last five medication-specific half-lives. The 17-item Hamilton Rating Scale for Depression (HRSD) (30) tool was used to gauge MDD severity. This instrument was administered by two board-certified psychiatrists, with patients exhibiting an HRSD score ≥ 17 being eligible for inclusion. Of the 71 patients included in this study, 22 exhibited anxiety-related features, 12 exhibited psychotic symptoms congruent with emotion, 6 exhibited psychotic symptoms not congruent with emotion, 14 had a history of suicidal thoughts, and 11 had a history of self-harm.

#### Healthy control recruitment

In total, 72 HCs that were age-, sex-, ethnicity-, education level-, and handedness-matched were recruited at random from the local community based on the national population register. The healthy status of HCs was confirmed through the use of the Structured Clinical Interview for DSM-IV Axis I disorders-Research version-Non-Patient Edition (SCID-I/NP).

### Magnetic resonance imaging acquisition and postprocessing

#### Image acquisition

All resting-state fMRI (rs-fMRI) scans were performed with a 3.0T General Electric scanner at the PET center of Renmin Hospital of Wuhan University using previously described methods (31). Briefly, participants were directed to lie in the supine position with their eyes closed while remaining awake and motionless. An echoplanar imaging (EPI) sequence was then conducted with the following settings: repetition time/echo time (TR/TE) 2000/30 ms, 32 slices, 64*64 matrix, 90° flip angle, 24 cm field of view, 3.0 mm slice thickness, no gap, and axial scanning 212 times for 16 min.

#### Data postprocessing

The Data Processing Assistant for rs-fMRI (DPARSF) (32) advanced edition based on SPM8 implemented in the MATLAB platform was used for all rs-fMRI data analyses. After discarding the first 5 imaging time points for each participant, those individuals exhibiting a maximal displacement > 2 mm in any direction (x, y, or z axis) or > 2° of maximal rotation were excluded following correction for head motion and slice timing. After spatial normalization to the MNI space and 3*3*3 mm^3^ resampling, images were smoothed using an 8 mm full width at half-maximum Gaussian kernel, subjected to bandpass filtration (0.01–0.1°Hz), and linearly detrended. Spurious covariates were additionally removed such as ventricular ROI signal, signal from a region centered in the white matter, and six head motion parameters obtained through rigid body correction.

#### Degree centrality calculations

Data processing assistant for rs-fMRI (DPARSF) was used for all DC calculations. As reported previously (33), Pearson’s correlation coefficients between a given voxel and all other voxels were used to establish a voxel-wise correlation matrix. Potential spurious connectivity was eliminated from this matrix through binarization for each correlation at an *r* > 0.25 threshold, thereby generating individual-level DC maps (34, 35) (Please see more details in Supplementary material). These maps were then normalized to Z-score maps *via* Fisher’s r-to-z transformation, and standard deviation within the whole gray matter mask (36), followed by spatial smoothing with a 6 mm full-width at half-maximum Gaussian kernel.

### Statistical analyses

SPSS was used for all statistical testing. Continuous and categorical data were compared between MDD and HC individuals *via* independent two-sample *t*-tests and chi-squared tests, with *P* < 0.05 as the threshold of significance. Individual whole-brain DC maps were subject to voxel-by-voxel ANCOVAs to detect group differences, with results being subject to GRF correction at the voxel level (*P* < 0.01), as GRF correction is better suited to this study than the false discovery rate (FDR; traditional approach with less smoothness) voxel-level familywise error (few; relatively conversive), and AlphaSim (less stringent) approaches (37, 38).

### Correlation analysis

Pearson’s correlations and multiple factors regression analysis were used to explore correlations between the severity of MDD and DC values in the six abnormal brain regions identified above and the demographic and clinical characteristics of patients included in this study.

### Classification analysis

An SVM approach conducted with the LIBSVM package in MATLAB was utilized to examine the ability of DC values in six abnormal regions of the brain (left lingual gyrus, left insula and right insula, right cerebellum, right superior parietal lobule, and left superior parietal lobule) to differentiate between MDD patients and HCs. The radial basis function (RBF) was selected as the kernel function, while a grid of parameters was evaluated with LIBSVM, and the optimal parameters including C (penalty coefficient) and g (gamma) were chosen. The accuracy values for all parameter settings were established, and the maximal cross-validation accuracy of these parameters was evaluated (Please see a more detailed description of the SVM method in Supplementary material).

## Results

### Participant characteristics

No significant differences in age, gender, or education level were evident when comparing MDD and HC participant groups (Table 1), while MDD patients exhibited higher HRSD-17 total scores relative to HCs, as expected.

**TABLE 1:** Demographic and clinical variables.

Demographic variables	Depression (70) A	Health control (71)
Age (mean/SD, year)	15.1 ± 1.9	16.5 ± 1.9
**Sex n(%)**		
Male	44 (62)	44 (61)
Female	27 (38)	28 (39)
**Education n(%)**		
High school or lower	65 (88.7)	63 (87.5)
Undergraduate	6 (11.3)	9 (12.5)
HRSD-17 total score (mean/SD)	22.9 (4.3)	8.7 (5.8)
Illness duration (mean/SD, month)	6.3 (3.6)	

### Group difference in degree centrality

**FIGURE 1:**
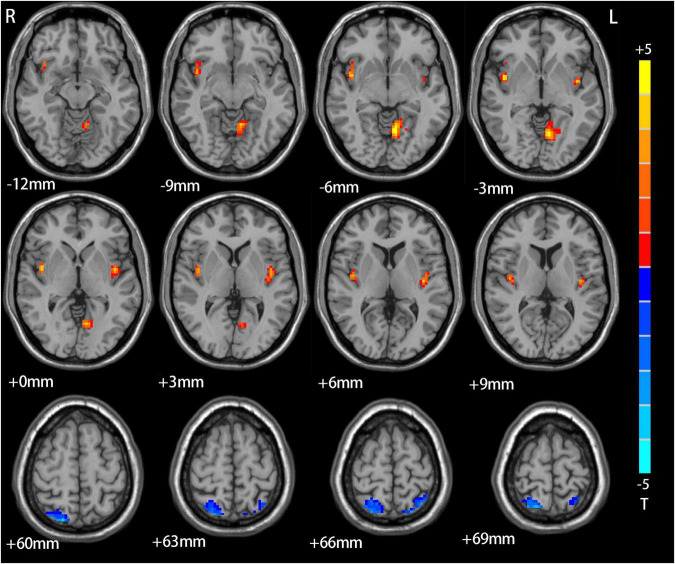
The difference between adolescents depression patients and HCs. Red means increase of DC value, blue means decrease of DC value.

Figure 1 summarizes significant differences in DC values between the adolescent MDD and HC groups. Relative to HC individuals, those with MDD present with higher DC in the left lingual gyrus, left insula, and right insula, as well as with decreased DC in the right cerebellum, right superior parietal lobule, and left superior parietal lobule (Table 2).

**TABLE 2:** Significant resting-state degree centrality difference across groups.

Clusterlocation adolescent depression vs. HC	Peak MNI coordinate			Number of voxels	*T*-value
	*x*	*y*	*z*		
Right cerebellum	30	−30	−39	199	−11.4
Left lingual	−9	−66	−3	105	9.5
Right insula	45	0	−3	74	9.2
Left insula	−42	−3	0	64	8.9
Right superior parietal	15	−63	72	157	−10.9
Left superior parietal	−18	−51	78	78	−9.8

DC, degree centrality; MNI, Montreal Neurological Institute.

### Correlation between degree centrality and HRSD-17 total scores

A matrix-based correlation approach and multiple factors regression analysis were used to explore relationships between DC values in the six abnormal brain regions identified above and the demographic and clinical characteristics of patients included in this study, but no significant correlations were detected between DC values and the severity of MDD symptoms (Please see Figure 1, Tables 1, 2 in Supplementary material).

### Support vector machine results

**FIGURE 2:**
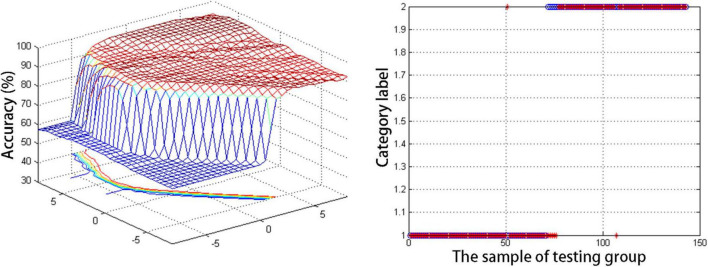
The general information of support vector machine results for discriminating adolescent depression patients and HCs.

Figure 2 summarizes the general SVM results when differentiating between MDD patients and HCs. Overall, abnormal DC values in the right cerebellum were found to offer the greatest utility when discriminating between these two participant groups, with respective accuracy, sensitivity, and specificity values of 95.1, 92.1, and 98.5%.

## Discussion

Here, DC values were explored as a promising neuroimaging biomarker capable of differentiating between adolescent MDD patients and healthy individuals. This approach revealed that DC maps for the right cerebellum were able to reliably discriminate between these two participant groups with respective accuracy, sensitivity, and specificity values of 95.1, 92.1, and 98.5%. Moreover, changes in DC values in the lingual area, insula, and superior parietal gyrus were evident when comparing adolescent MDD patients and HCs. However, no correlations were observed between abnormal DC values and clinical findings in patients.

The accuracy value detected herein when using DC values to differentiate between MDD and HC individuals (95.1%) is similar to the 94% value published in a recent report employing DC maps to differentiate between healthy adults and MDD patients (12), and both values are much higher than those for other previously reported neuroimaging modalities, which generally range from 26 to 81% (39–41). These results underscore the value of DC maps as tools capable of accurately extracting abnormal connectivity findings specific to a given illness, although follow-up validation of these results remains to be completed. Depression is regarded as an affective disorder that can impact individuals of all ages, but the rate of diagnosis among younger individuals has been rising in recent years (3). Sub-clinical depressive symptom incidence is also a common finding in healthy adolescent individuals (42), highlighting the value of using DC maps to differentiate between potentially at-risk adolescents who may be suffering from sub-clinical depression and healthy peers.

Here, reductions in DC values were observed in both the right cerebellum and bilateral superior parietal regions in adolescents with MDD relative to HC individuals. In addition to serving as the motor domain, the cerebellum is important for many cognitive and affective functions (43). Cerebellar lesions, accordingly, can result in affective and/or cognitive symptoms that are collectively referred to as cerebellar cognitive affective syndrome, which results in characteristically impaired linguistic and executive function with concomitant apathy, depressed mood, or abnormal social cognition (44). The observed loss of cerebellar DC in this study is consistent with recent evidence from analyses of both first-episode treatment-naïve adolescent MDD patients and unmedicated adults with this disease (45). Specifically, other studies have reported an increase in the connectivity of the left cerebellum in depressed adolescents considered suicidal, whereas non-suicidal adolescent MDD patients exhibited decreased connectivity in the lower left cerebellum, potentially owing to differences in sample subgrouping in these studies. The results of the present analysis are consistent with prior data regarding the important role that the cerebellum plays in the pathogenesis of MDD (46–49), highlighting the possibility of defining novel neuroimaging-based biomarkers for this condition.

The superior parietal lobule (SPL) is an important mediator of diverse perceptive, cognitive, and motor-associated processes such as spatial cognition, attention, working memory, visual perception, and visually guided visuomotor functions (50, 51). The ENIGMA-MDD working group performed a meta-analysis which revealed that relative to HCs, depressed adolescents exhibited significant reductions in the surface area of the left (213 cases, 294 controls) and right (213 cases, 293 controls) superior parietal cortex (52). Depressed children and adolescents who were naïve to psychotropic medication treatment also exhibited a thinner SPL than that observed in matched HC individuals (53). Here, lower DC in the bilateral SPL was observed in adolescent MDD patients, in line with prior evidence from adult MDD patients (54, 55). In one prior fMRI-based study employing attentional tasks, medication-naive first-episode adolescent MDD patients performed more poorly in an attention-focused testing battery, and exhibited lower levels of parietal lobe activation in regions related to tasks being performed for both attentional switching (Switch task) and error detection (Stop task) (54). Prior work also suggests that medication-naïve adolescent MDD patients exhibit changes in brain function compared to those in adults with MDD. Changes in SPL function may thus represent an early pathogenic abnormality associated with the onset of MDD, although more work will be necessary to test this possibility.

This study further revealed that increases in DC values were evident in the lingual gyrus in adolescents with MDD. Lingual gyrus activation has previously been reported to be evident in the context of tasks requiring memorization and the maintenance of human faces in the working memory (55), and it also serves as a structural component of the visual cortex that is critical for word identification and recognition (56). It is also potentially functionally linked with the amygdala, and may thus play a role in emotional processing (57). The ENIGMA-MDD group meta-analysis reported the left lingual gyrus surface area to be significantly reduced in adolescents with depression (58). While this meta-analysis incorporated medicated patients and failed to correct for total surface area, lingual gray matter density was found to predict patient responses to antidepressant treatment. Another recent meta-analysis of the neurological activity of youths and adults diagnosed with MDD in the context of emotional processing indicated that lingual gyrus activity levels were higher in youths with depression as compared to adults (13). Accordingly, the finding herein that the DC of the left lingual gyrus was increased in adolescent MDD patients may be consistent with intrinsic functional alterations in this area in adolescents with depression.

Here, increases in bilateral insular DC values were detected in MDD patients relative to HCs. The insula serves as a site of fronto-limbic network integration owing to its anatomical connections to associated brain regions (59). Both adolescent and adult MDD patients consistently exhibit abnormal fronto-limbic network activity, with a bias toward negative stimuli and increased attention to and processing of emotional information (60–62). In line with the present results, prior rs-fMRI studies have found adolescents with MDD to exhibit increases in the activity and functional connectivity of the insula (63–65). Studies have also reported hypoactivation of the dorsal anterior insula in executive function tasks and hypoactivation of the posterior insula in positive valence tasks in neuroimaging studies (10). Insular structural alterations have also been reported in adolescents with MDD in the meta-analysis published by the ENIGMA-MDD group (44). These results suggest that insular dysfunction may represent a mechanism underlying the pathophysiological basis for MDD in adolescents. Notably, reductions in insular functional connectivity have been reported in adolescents with depression suffering from comorbid anxiety disorder, ADHD, and post-traumatic stress disorder (66, 67), consistent with the multidisciplinary function that the insula plays in a range of psychiatric conditions (66–68). However, additional studies with appropriate patient subgroups will be needed to test these possibilities.

There are multiple limitations to this analysis. For one, the lack of any observed correlation between DC values and MDD symptoms may be impacted by a range of factors including the timing of DC changes, cognitive impairment, and emotional/somatic disease-related burden. As the present study relied on rs-fMRI data in the absence of any emotion- or cognition-related tasks, additional follow-up studies exploring DC abnormalities in these contexts are warranted for adolescents with MDD. This study also included a relatively small number of patients, potentially contributing to this lack of any observed correlation. Moreover, accurately subgrouping MDD patients based on their comorbidities (such as anxiety, attention deficit-hyperactivity disorder, or stress-associated disorders) or clinical findings may have an impact on these results, highlighting a need for more detailed subgroup-based analyses (69–72). It is also important to take into consideration that some adolescents with BPD are initially misdiagnosed with MDD, and the delay between the initial onset of affective symptoms and the diagnosis of BPD can be up to 10 years (73). As such, further follow-up validation of these results will be critical. This study also specifically focused on patients with an illness duration of < 12 months to ensure that patients were able to accurately recall depressive episodes. However, this inclusion criterion has the potential to have biased these results. Lastly, while the HDRS has been used as a gold standard tool to rate the severity of MDD in adolescents, there have been questions raised in recent years regarding its internal reliability, its discriminant and convergent validity, and its utility when assessing adolescent patients. As such, the establishment of diagnostic tools more closely tailored to the symptoms of adolescent MDD may be of value in future research.

In summary, the present results suggest that adolescent MDD is associated with DC abnormalities in several brain regions associated with sensorimotor activity, emotional processing, and cognitive impairment. These DC values may ultimately offer good utility as a neuroimaging biomarker for the early detection and monitoring of MDD in this patient population.

## Data availability statement

The raw data supporting the conclusions of this article will be made available by the authors, without undue reservation.

## Ethics statement

The studies involving human participants were reviewed and approved by Renmin Hospital of Wuhan University (Wuhan, China). Written informed consent to participate in this study was provided by the participants’ legal guardian/next of kin.

## Author contributions

All authors listed have made a substantial, direct, and intellectual contribution to the work, and approved it for publication.
