# Supplementary material for: Abnormal degree centrality in first-episode medication-free adolescent depression at rest: A functional magnetic resonance imaging study and support vector machine analysis
Source: Front Psychiatry. 2022 Sep 29;13:926292. doi: 10.3389/fpsyt.2022.926292 (PMC9556654; doi:10.3389/fpsyt.2022.926292)
Supplement: Supplementary file 1 [file Data_Sheet_1.pdf]

Supplementary Material

1 Supplementary Methods

Image Acquisition

An Achieva 3TMRI scanner (Philips, Netherlands) was utilized for resting-state functional magnetic resonance imaging (rsfMRI). Patients were asked to lie down and close their eyes but remain awake. A prototype quadrature birdcage head coil filled with foam was used to minimize head movement. Functional imaging had the following parameters: ratio of repetition time to echo time (TR/TE) (2,000/30 ms), slice thickness (5 mm), pitch (1 mm), field of view (220 × 220 mm) and flip angle (90◦). On the structural scan (T1-weighted), the following settings were used: spin-echo sequence, repetition time (TR) = 20 ms, echo time (TE) = 3.5 ms, slice thickness = 1 mm, and field of view (FOV) = 220 × 220 mm.

Data Preprocessing

Imaging data of rs-fMRI were preprocessed by using DPARSF software in MATLAB. The first 5 time points were removed. Slice time and head motion were corrected. No participants had more than 2mm of maximal displacement in x, y, or z axis and more than 2° of maximal rotation. The structure of each patient was registered to its functional image. The structure of each patient was divided, and a template was created to normalize the structures of the patients after they were defined according to the Montreal Neurological Institute (MNI) standard template, the standardization process of the spatial deformation of the modulation and the structure of the voxel size using $1 \times 1 \times 1\ mm^3$. Finally, the use of the structure of each patient to the function of the conversion matrix was also standardized to the MNI space. During the process of functional image normalization, head motion parameters, white matter signal, and cerebrospinal fluid signal were used as removal covariates , and voxel size of $3 \times 3 \times 3\ mm^3$ was used as functional covariate. The obtained images were subsequently smoothed with an 8mm full width at half-maximum Gaussian kernel, band pass filtered (0.01–0.1Hz), and linearly detrended to lessen the effect of low-frequency drifts and physiologic highfrequency noise. Several spurious covariates were removed, including signal from a region centered in the white matter, 6 head motion parameters obtained by rigid body correction, and signal from a ventricular ROI. The global signal removal may introduce artifacts into the data and distort resting-state connectivity patterns. Furthermore, the regression of the global signal may significantly distort results when studying clinical populations. Therefore, the global signal was preserved.

DC calculation

DC Calculation DC measure was calculated by using DPARSF. In line with our previous study, [1]a voxel-wise correlation matrix was firstly built by computing the Pearson’s correlation coefficients between a selected voxel and all other voxels. Accorrding to previous studies, From the n × n Pearson's correlation coefficient matrix, a map of the degree of the connectivity was computed by counting for each voxel the number of voxels it was correlated to above a threshold of r > 0.25. A high threshold was chosen to eliminate counting voxels that had low temporal correlation attributable to signal noise. Different threshold selections did not qualitatively change the results for cortex.[2-3] To eliminate potential spurious connectivity, the correlation matrix was binarized by thresholding at r>0.25 for each correlation to generate a individual-level DC map. Then the DC map was normalized into a Z-score map by using the Fisher’s r-to-z transformation, [4]and standard deviation within the whole gray matter

mask,[5]and spatially smoothed by using a 6mm full-width at half maximum Gaussian kernel.

Classification Analysis

We use LibSVM method based on Weka. LibSVM is a library about the SVM developed by Professor Lin et al. in 2001. It has been widely used in bioinformatics. It has the advantages of being a small program that is flexible, with less inputting parameters, is open source to expand easily, and thus has become the most widely used SVM Library in China. This library tool can be accessed at https://www.csie.ntu.edu.tw/~cjlin/. Weka is a free and noncommercial mining platform, which has a series of functional modules that basically meet various needs in data analysis, such as a variety of different classification and regression algorithms and performing cross validation during classification, automatically. LibSVM classification has been supported since Weka version 3.5.

Considering most neuroimaging studies are likely to be nonlinear, kernel SVM has been proposed to achieve better performance than the other methods for nonlinear depression classification. [2] In this experiment, the radial basis function (RBF) is adopted as the kernel function, which is also the default setting in LibSVM. Two parameters, the cost (c) and gamma (g), need to be determined before building the classification model by using Weka. The parameter c is called the penalty coefficient. The higher the value of c is, the easier it is to over fit. And g is a parameter of RBF function after it is selected as kernel which affects the speed of process of training and prediction. There is no universally recognized best method for parameter selection, and the common method is to let c and g take values within a certain range, and then set different c and g in the process of training set data classification. Finally, use cross validation to get the classification accuracy verified by the training set in this groups c and g, and select the group with the best classification result by comparison. It is a complicated process, but in LibSVM toolkit, the parameter optimization is automated, and it no longer needs to be manually adjusted. We use the program, grid.py in the LibSVM tool folder to get the optimal parameters.

2 Supplementary Results

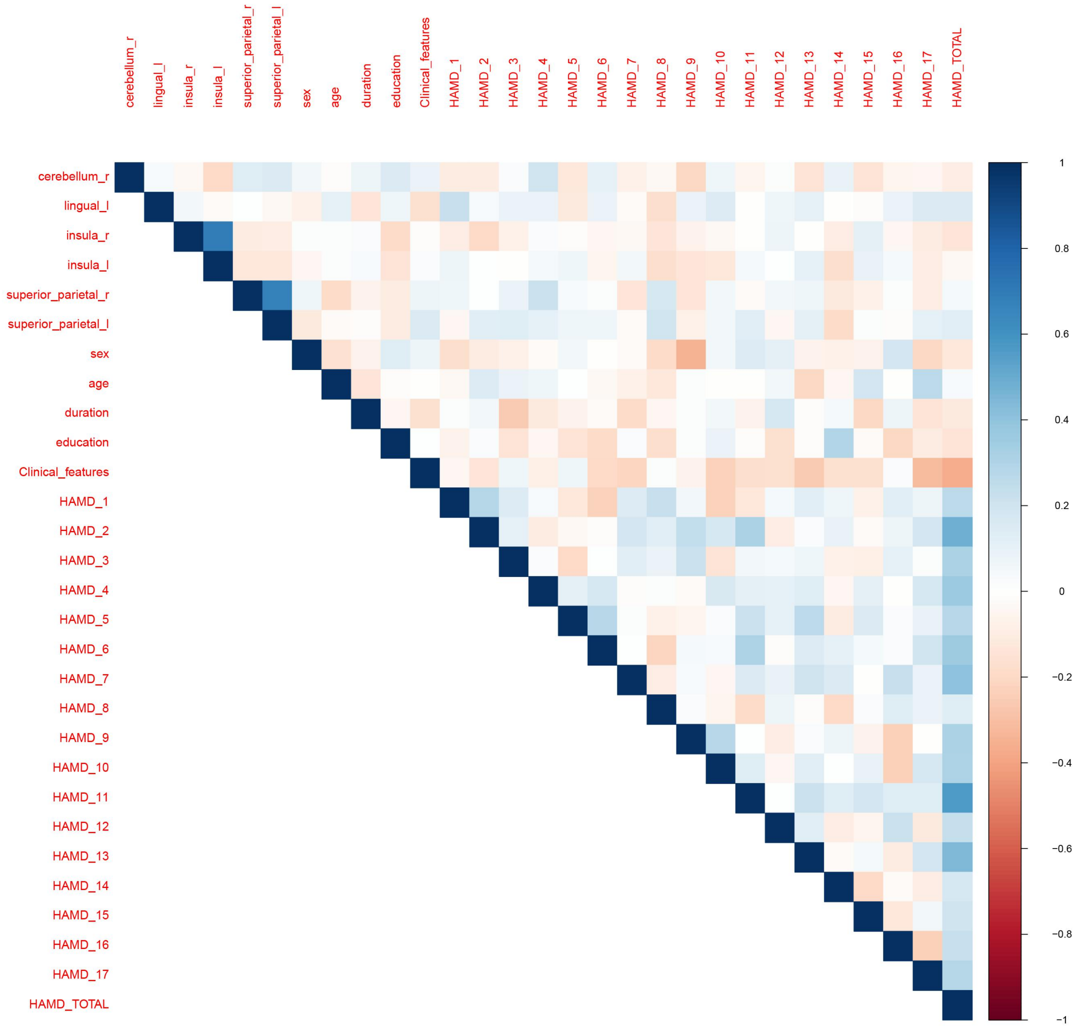

eFigure1. Heatmap of correlation between Correlation analysis of DC vaule and demographic and clinical factors. P<0.05 was taken as statistical significant. No significant correlation was found.

eTable1. Correlation analysis of DC vaule and demographic and clinical factors

	row	column	cor	p
1	cerebellum_r	lingual_l	0.03	0.78
2	cerebellum_r	insula_r	-0.03	0.80
3	lingual_l	insula_r	0.05	0.65
4	cerebellum_r	insula_l	-0.20	0.10
5	lingual_l	insula_l	-0.03	0.82
6	insula_r	insula_l	0.70	0.00
7	cerebellum_r	superior_parietal_r	0.13	0.26
8	lingual_l	superior_parietal_r	0.01	0.93
9	insula_r	superior_parietal_r	-0.10	0.40
10	insula_l	superior_parietal_r	-0.13	0.30
11	cerebellum_r	superior_parietal_l	0.16	0.19

12	lingual_l	superior_parietal_l	-0.04	0.76
13	insula_r	superior_parietal_l	-0.10	0.42
14	insula_l	superior_parietal_l	-0.13	0.29
15	superior_parietal_r	superior_parietal_l	0.68	0.00
16	cerebellum_r	sex	0.06	0.63
17	lingual_l	sex	-0.08	0.52
18	insula_r	sex	0.01	0.91
19	insula_l	sex	-0.05	0.69
20	superior_parietal_r	sex	0.06	0.61
21	superior_parietal_l	sex	-0.11	0.35
22	cerebellum_r	age	-0.01	0.91
23	lingual_l	age	0.10	0.40
24	insula_r	age	0.02	0.88
25	insula_l	age	0.02	0.89
26	superior_parietal_r	age	-0.19	0.12
27	superior_parietal_l	age	-0.03	0.81
28	sex	age	-0.15	0.21
29	cerebellum_r	duration	0.08	0.52
30	lingual_l	duration	-0.13	0.28
31	insula_r	duration	0.03	0.82
32	insula_l	duration	0.03	0.78
33	superior_parietal_r	duration	-0.07	0.58
34	superior_parietal_l	duration	-0.02	0.90
35	sex	duration	-0.06	0.62
36	age	duration	-0.14	0.26
37	cerebellum_r	education	0.16	0.19
38	lingual_l	education	0.07	0.57
39	insula_r	education	-0.18	0.12
40	insula_l	education	-0.15	0.22
41	superior_parietal_r	education	-0.11	0.37
42	superior_parietal_l	education	-0.11	0.38
43	sex	education	0.13	0.27
44	age	education	-0.01	0.91
45	duration	education	-0.04	0.72
46	cerebellum_r	Clinical_features	0.08	0.48
47	lingual_l	Clinical_features	-0.17	0.16
48	insula_r	Clinical_features	-0.02	0.87
49	insula_l	Clinical_features	0.02	0.87
50	superior_parietal_r	Clinical_features	0.07	0.55
51	superior_parietal_l	Clinical_features	0.16	0.18
52	sex	Clinical_features	0.08	0.52
53	age	Clinical_features	0.00	0.97
54	duration	Clinical_features	-0.17	0.16
55	education	Clinical_features	0.00	0.99

56	cerebellum_r	HAMD_1	-0.10	0.39
57	lingual_l	HAMD_1	0.22	0.06
58	insula_r	HAMD_1	-0.09	0.44
59	insula_l	HAMD_1	0.08	0.52
60	superior_parietal_r	HAMD_1	0.07	0.59
61	superior_parietal_l	HAMD_1	-0.05	0.71
62	sex	HAMD_1	-0.18	0.14
63	age	HAMD_1	-0.04	0.75
64	duration	HAMD_1	0.01	0.90
65	education	HAMD_1	-0.06	0.62
66	Clinical_features	HAMD_1	-0.05	0.68
67	cerebellum_r	HAMD_2	-0.10	0.39
68	lingual_l	HAMD_2	0.04	0.75
69	insula_r	HAMD_2	-0.20	0.10
70	insula_l	HAMD_2	0.01	0.96
71	superior_parietal_r	HAMD_2	0.00	0.99
72	superior_parietal_l	HAMD_2	0.12	0.30
73	sex	HAMD_2	-0.11	0.38
74	age	HAMD_2	0.14	0.24
75	duration	HAMD_2	0.06	0.63
76	education	HAMD_2	0.02	0.87
77	Clinical_features	HAMD_2	-0.14	0.25
78	HAMD_1	HAMD_2	0.28	0.02
79	cerebellum_r	HAMD_3	0.03	0.82
80	lingual_l	HAMD_3	0.08	0.49
81	insula_r	HAMD_3	-0.07	0.54
82	insula_l	HAMD_3	0.00	0.99
83	superior_parietal_r	HAMD_3	0.08	0.48
84	superior_parietal_l	HAMD_3	0.14	0.25
85	sex	HAMD_3	-0.07	0.55
86	age	HAMD_3	0.08	0.51
87	duration	HAMD_3	-0.25	0.03
88	education	HAMD_3	-0.14	0.24
89	Clinical_features	HAMD_3	0.06	0.59
90	HAMD_1	HAMD_3	0.15	0.22
91	HAMD_2	HAMD_3	0.10	0.39
92	cerebellum_r	HAMD_4	0.20	0.10
93	lingual_l	HAMD_4	0.08	0.48
94	insula_r	HAMD_4	0.02	0.85
95	insula_l	HAMD_4	0.04	0.74
96	superior_parietal_r	HAMD_4	0.22	0.07
97	superior_parietal_l	HAMD_4	0.10	0.38
98	sex	HAMD_4	-0.02	0.87
99	age	HAMD_4	0.07	0.58

100	duration	HAMD_4	-0.12	0.32
101	education	HAMD_4	-0.05	0.70
102	Clinical_features	HAMD_4	-0.09	0.46
103	HAMD_1	HAMD_4	0.04	0.77
104	HAMD_2	HAMD_4	-0.11	0.38
105	HAMD_3	HAMD_4	0.03	0.83
106	cerebellum_r	HAMD_5	-0.13	0.29
107	lingual_l	HAMD_5	-0.12	0.34
108	insula_r	HAMD_5	-0.02	0.89
109	insula_l	HAMD_5	0.06	0.60
110	superior_parietal_r	HAMD_5	0.04	0.77
111	superior_parietal_l	HAMD_5	0.07	0.57
112	sex	HAMD_5	0.05	0.67
113	age	HAMD_5	0.00	0.99
114	duration	HAMD_5	-0.07	0.58
115	education	HAMD_5	-0.15	0.22
116	Clinical_features	HAMD_5	0.06	0.62
117	HAMD_1	HAMD_5	-0.13	0.29
118	HAMD_2	HAMD_5	-0.03	0.79
119	HAMD_3	HAMD_5	-0.20	0.10
120	HAMD_4	HAMD_5	0.12	0.32
121	cerebellum_r	HAMD_6	0.10	0.40
122	lingual_l	HAMD_6	0.09	0.47
123	insula_r	HAMD_6	-0.04	0.72
124	insula_l	HAMD_6	-0.05	0.65
125	superior_parietal_r	HAMD_6	0.01	0.93
126	superior_parietal_l	HAMD_6	0.06	0.60
127	sex	HAMD_6	-0.01	0.96
128	age	HAMD_6	-0.04	0.76
129	duration	HAMD_6	-0.03	0.81
130	education	HAMD_6	-0.19	0.12
131	Clinical_features	HAMD_6	-0.20	0.10
132	HAMD_1	HAMD_6	-0.22	0.06
133	HAMD_2	HAMD_6	-0.02	0.90
134	HAMD_3	HAMD_6	0.00	0.99
135	HAMD_4	HAMD_6	0.17	0.15
136	HAMD_5	HAMD_6	0.27	0.02
137	cerebellum_r	HAMD_7	-0.07	0.53
138	lingual_l	HAMD_7	-0.02	0.85
139	insula_r	HAMD_7	-0.04	0.74
140	insula_l	HAMD_7	0.05	0.66
141	superior_parietal_r	HAMD_7	-0.14	0.25
142	superior_parietal_l	HAMD_7	-0.03	0.81
143	sex	HAMD_7	-0.02	0.86

144	age	HAMD_7	-0.07	0.56
145	duration	HAMD_7	-0.18	0.13
146	education	HAMD_7	0.02	0.84
147	Clinical_features	HAMD_7	-0.21	0.08
148	HAMD_1	HAMD_7	0.14	0.23
149	HAMD_2	HAMD_7	0.18	0.13
150	HAMD_3	HAMD_7	0.13	0.30
151	HAMD_4	HAMD_7	-0.01	0.92
152	HAMD_5	HAMD_7	0.02	0.88
153	HAMD_6	HAMD_7	0.01	0.94
154	cerebellum_r	HAMD_8	-0.04	0.75
155	lingual_l	HAMD_8	-0.18	0.14
156	insula_r	HAMD_8	-0.13	0.28
157	insula_l	HAMD_8	-0.18	0.14
158	superior_parietal_r	HAMD_8	0.18	0.13
159	superior_parietal_l	HAMD_8	0.20	0.09
160	sex	HAMD_8	-0.19	0.12
161	age	HAMD_8	-0.13	0.30
162	duration	HAMD_8	-0.05	0.69
163	education	HAMD_8	-0.18	0.14
164	Clinical_features	HAMD_8	0.02	0.90
165	HAMD_1	HAMD_8	0.23	0.05
166	HAMD_2	HAMD_8	0.12	0.31
167	HAMD_3	HAMD_8	0.09	0.47
168	HAMD_4	HAMD_8	0.01	0.93
169	HAMD_5	HAMD_8	-0.07	0.55
170	HAMD_6	HAMD_8	-0.21	0.07
171	HAMD_7	HAMD_8	-0.10	0.41
172	cerebellum_r	HAMD_9	-0.21	0.08
173	lingual_l	HAMD_9	0.09	0.46
174	insula_r	HAMD_9	-0.06	0.59
175	insula_l	HAMD_9	-0.13	0.28
176	superior_parietal_r	HAMD_9	-0.13	0.28
177	superior_parietal_l	HAMD_9	-0.08	0.52
178	sex	HAMD_9	-0.34	0.00
179	age	HAMD_9	0.01	0.92
180	duration	HAMD_9	0.02	0.88
181	education	HAMD_9	0.02	0.87
182	Clinical_features	HAMD_9	-0.06	0.61
183	HAMD_1	HAMD_9	0.06	0.63
184	HAMD_2	HAMD_9	0.25	0.04
185	HAMD_3	HAMD_9	0.22	0.07
186	HAMD_4	HAMD_9	-0.02	0.87
187	HAMD_5	HAMD_9	-0.06	0.64

188	HAMD_6	HAMD_9	0.04	0.71
189	HAMD_7	HAMD_9	0.03	0.78
190	HAMD_8	HAMD_9	0.02	0.86
191	cerebellum_r	HAMD_10	0.07	0.57
192	lingual_l	HAMD_10	0.15	0.21
193	insula_r	HAMD_10	-0.04	0.77
194	insula_l	HAMD_10	-0.13	0.30
195	superior_parietal_r	HAMD_10	0.06	0.63
196	superior_parietal_l	HAMD_10	0.06	0.64
197	sex	HAMD_10	0.05	0.66
198	age	HAMD_10	0.00	0.98
199	duration	HAMD_10	0.06	0.63
200	education	HAMD_10	0.08	0.49
201	Clinical_features	HAMD_10	-0.23	0.05
202	HAMD_1	HAMD_10	-0.22	0.06
203	HAMD_2	HAMD_10	0.18	0.13
204	HAMD_3	HAMD_10	-0.14	0.23
205	HAMD_4	HAMD_10	0.17	0.17
206	HAMD_5	HAMD_10	0.02	0.87
207	HAMD_6	HAMD_10	0.04	0.77
208	HAMD_7	HAMD_10	-0.04	0.73
209	HAMD_8	HAMD_10	-0.05	0.66
210	HAMD_9	HAMD_10	0.27	0.02
211	cerebellum_r	HAMD_11	-0.06	0.63
212	lingual_l	HAMD_11	0.00	0.99
213	insula_r	HAMD_11	-0.01	0.94
214	insula_l	HAMD_11	0.04	0.74
215	superior_parietal_r	HAMD_11	-0.02	0.86
216	superior_parietal_l	HAMD_11	0.12	0.31
217	sex	HAMD_11	0.15	0.23
218	age	HAMD_11	0.00	0.99
219	duration	HAMD_11	-0.06	0.59
220	education	HAMD_11	-0.01	0.92
221	Clinical_features	HAMD_11	-0.17	0.15
222	HAMD_1	HAMD_11	-0.12	0.31
223	HAMD_2	HAMD_11	0.32	0.01
224	HAMD_3	HAMD_11	0.06	0.64
225	HAMD_4	HAMD_11	0.11	0.34
226	HAMD_5	HAMD_11	0.22	0.07
227	HAMD_6	HAMD_11	0.30	0.01
228	HAMD_7	HAMD_11	0.16	0.20
229	HAMD_8	HAMD_11	-0.18	0.13
230	HAMD_9	HAMD_11	0.01	0.96
231	HAMD_10	HAMD_11	0.14	0.26

232	cerebellum_r	HAMD_12	0.02	0.88
233	lingual_l	HAMD_12	0.06	0.61
234	insula_r	HAMD_12	0.07	0.55
235	insula_l	HAMD_12	0.01	0.95
236	superior_parietal_r	HAMD_12	0.06	0.62
237	superior_parietal_l	HAMD_12	-0.04	0.71
238	sex	HAMD_12	0.12	0.34
239	age	HAMD_12	0.05	0.66
240	duration	HAMD_12	0.17	0.15
241	education	HAMD_12	-0.17	0.16
242	Clinical_features	HAMD_12	-0.16	0.17
243	HAMD_1	HAMD_12	0.04	0.73
244	HAMD_2	HAMD_12	-0.10	0.41
245	HAMD_3	HAMD_12	0.05	0.71
246	HAMD_4	HAMD_12	0.10	0.39
247	HAMD_5	HAMD_12	0.10	0.39
248	HAMD_6	HAMD_12	-0.02	0.89
249	HAMD_7	HAMD_12	0.10	0.43
250	HAMD_8	HAMD_12	0.08	0.51
251	HAMD_9	HAMD_12	-0.10	0.43
252	HAMD_10	HAMD_12	-0.04	0.72
253	HAMD_11	HAMD_12	0.01	0.94
254	cerebellum_r	HAMD_13	-0.15	0.23
255	lingual_l	HAMD_13	0.11	0.38
256	insula_r	HAMD_13	-0.01	0.94
257	insula_l	HAMD_13	0.10	0.39
258	superior_parietal_r	HAMD_13	0.06	0.60
259	superior_parietal_l	HAMD_13	0.12	0.32
260	sex	HAMD_13	-0.07	0.56
261	age	HAMD_13	-0.20	0.09
262	duration	HAMD_13	-0.01	0.91
263	education	HAMD_13	-0.02	0.87
264	Clinical_features	HAMD_13	-0.26	0.03
265	HAMD_1	HAMD_13	0.12	0.32
266	HAMD_2	HAMD_13	0.02	0.85
267	HAMD_3	HAMD_13	0.07	0.57
268	HAMD_4	HAMD_13	0.13	0.27
269	HAMD_5	HAMD_13	0.26	0.03
270	HAMD_6	HAMD_13	0.15	0.23
271	HAMD_7	HAMD_13	0.21	0.08
272	HAMD_8	HAMD_13	-0.02	0.88
273	HAMD_9	HAMD_13	0.02	0.85
274	HAMD_10	HAMD_13	0.12	0.30
275	HAMD_11	HAMD_13	0.22	0.07

276	HAMD_12	HAMD_13	0.12	0.30
277	cerebellum_r	HAMD_14	0.10	0.42
278	lingual_l	HAMD_14	0.00	0.99
279	insula_r	HAMD_14	-0.10	0.41
280	insula_l	HAMD_14	-0.17	0.15
281	superior_parietal_r	HAMD_14	-0.11	0.35
282	superior_parietal_l	HAMD_14	-0.18	0.13
283	sex	HAMD_14	-0.08	0.51
284	age	HAMD_14	-0.05	0.68
285	duration	HAMD_14	0.04	0.74
286	education	HAMD_14	0.30	0.01
287	Clinical_features	HAMD_14	-0.17	0.17
288	HAMD_1	HAMD_14	0.07	0.58
289	HAMD_2	HAMD_14	0.10	0.41
290	HAMD_3	HAMD_14	-0.07	0.55
291	HAMD_4	HAMD_14	-0.04	0.73
292	HAMD_5	HAMD_14	-0.11	0.37
293	HAMD_6	HAMD_14	0.11	0.38
294	HAMD_7	HAMD_14	0.15	0.20
295	HAMD_8	HAMD_14	-0.19	0.11
296	HAMD_9	HAMD_14	0.07	0.54
297	HAMD_10	HAMD_14	0.01	0.95
298	HAMD_11	HAMD_14	0.13	0.27
299	HAMD_12	HAMD_14	-0.10	0.42
300	HAMD_13	HAMD_14	-0.02	0.87
301	cerebellum_r	HAMD_15	-0.14	0.24
302	lingual_l	HAMD_15	-0.01	0.91
303	insula_r	HAMD_15	0.12	0.33
304	insula_l	HAMD_15	0.10	0.42
305	superior_parietal_r	HAMD_15	-0.08	0.51
306	superior_parietal_l	HAMD_15	0.01	0.91
307	sex	HAMD_15	-0.07	0.59
308	age	HAMD_15	0.19	0.12
309	duration	HAMD_15	-0.21	0.08
310	education	HAMD_15	-0.03	0.81
311	Clinical_features	HAMD_15	-0.16	0.18
312	HAMD_1	HAMD_15	-0.07	0.56
313	HAMD_2	HAMD_15	-0.02	0.86
314	HAMD_3	HAMD_15	-0.09	0.47
315	HAMD_4	HAMD_15	0.11	0.36
316	HAMD_5	HAMD_15	0.15	0.21
317	HAMD_6	HAMD_15	0.05	0.70
318	HAMD_7	HAMD_15	0.00	1.00
319	HAMD_8	HAMD_15	0.03	0.82

320	HAMD_9	HAMD_15	-0.06	0.62
321	HAMD_10	HAMD_15	0.09	0.45
322	HAMD_11	HAMD_15	0.18	0.12
323	HAMD_12	HAMD_15	-0.05	0.68
324	HAMD_13	HAMD_15	0.04	0.71
325	HAMD_14	HAMD_15	-0.20	0.10
326	cerebellum_r	HAMD_16	-0.05	0.65
327	lingual_l	HAMD_16	0.08	0.50
328	insula_r	HAMD_16	-0.05	0.70
329	insula_l	HAMD_16	0.05	0.70
330	superior_parietal_r	HAMD_16	0.01	0.91
331	superior_parietal_l	HAMD_16	-0.02	0.88
332	sex	HAMD_16	0.18	0.12
333	age	HAMD_16	0.00	0.98
334	duration	HAMD_16	0.08	0.53
335	education	HAMD_16	-0.21	0.08
336	Clinical_features	HAMD_16	0.02	0.84
337	HAMD_1	HAMD_16	0.13	0.29
338	HAMD_2	HAMD_16	0.07	0.54
339	HAMD_3	HAMD_16	0.10	0.40
340	HAMD_4	HAMD_16	0.00	0.99
341	HAMD_5	HAMD_16	0.02	0.86
342	HAMD_6	HAMD_16	0.02	0.84
343	HAMD_7	HAMD_16	0.22	0.06
344	HAMD_8	HAMD_16	0.13	0.27
345	HAMD_9	HAMD_16	-0.23	0.05
346	HAMD_10	HAMD_16	-0.23	0.05
347	HAMD_11	HAMD_16	0.14	0.26
348	HAMD_12	HAMD_16	0.22	0.07
349	HAMD_13	HAMD_16	-0.10	0.40
350	HAMD_14	HAMD_16	-0.02	0.86
351	HAMD_15	HAMD_16	-0.12	0.30
352	cerebellum_r	HAMD_17	-0.04	0.73
353	lingual_l	HAMD_17	0.15	0.20
354	insula_r	HAMD_17	-0.11	0.37
355	insula_l	HAMD_17	-0.09	0.45
356	superior_parietal_r	HAMD_17	-0.10	0.41
357	superior_parietal_l	HAMD_17	0.10	0.40
358	sex	HAMD_17	-0.20	0.09
359	age	HAMD_17	0.27	0.02
360	duration	HAMD_17	-0.14	0.23
361	education	HAMD_17	-0.10	0.39
362	Clinical_features	HAMD_17	-0.32	0.01
363	HAMD_1	HAMD_17	0.08	0.52

364	HAMD_2	HAMD_17	0.18	0.12
365	HAMD_3	HAMD_17	0.02	0.88
366	HAMD_4	HAMD_17	0.18	0.14
367	HAMD_5	HAMD_17	0.09	0.45
368	HAMD_6	HAMD_17	0.20	0.10
369	HAMD_7	HAMD_17	0.08	0.51
370	HAMD_8	HAMD_17	0.08	0.50
371	HAMD_9	HAMD_17	0.00	1.00
372	HAMD_10	HAMD_17	0.17	0.16
373	HAMD_11	HAMD_17	0.14	0.25
374	HAMD_12	HAMD_17	-0.12	0.34
375	HAMD_13	HAMD_17	0.19	0.12
376	HAMD_14	HAMD_17	-0.09	0.45
377	HAMD_15	HAMD_17	0.06	0.62
378	HAMD_16	HAMD_17	-0.23	0.05
379	cerebellum_r	HAMD_TOTAL	-0.10	0.42
380	lingual_l	HAMD_TOTAL	0.16	0.19
381	insula_r	HAMD_TOTAL	-0.13	0.28
382	insula_l	HAMD_TOTAL	-0.04	0.76
383	superior_parietal_r	HAMD_TOTAL	0.05	0.69
384	superior_parietal_l	HAMD_TOTAL	0.13	0.29
385	sex	HAMD_TOTAL	-0.12	0.32
386	age	HAMD_TOTAL	0.04	0.75
387	duration	HAMD_TOTAL	-0.12	0.32
388	education	HAMD_TOTAL	-0.14	0.23
389	Clinical_features	HAMD_TOTAL	-0.36	0.00
390	HAMD_1	HAMD_TOTAL	0.26	0.03
391	HAMD_2	HAMD_TOTAL	0.48	0.00
392	HAMD_3	HAMD_TOTAL	0.32	0.01
393	HAMD_4	HAMD_TOTAL	0.37	0.00
394	HAMD_5	HAMD_TOTAL	0.27	0.02
395	HAMD_6	HAMD_TOTAL	0.35	0.00
396	HAMD_7	HAMD_TOTAL	0.40	0.00
397	HAMD_8	HAMD_TOTAL	0.14	0.25
398	HAMD_9	HAMD_TOTAL	0.32	0.01
399	HAMD_10	HAMD_TOTAL	0.30	0.01
400	HAMD_11	HAMD_TOTAL	0.56	0.00
401	HAMD_12	HAMD_TOTAL	0.24	0.05
402	HAMD_13	HAMD_TOTAL	0.44	0.00
403	HAMD_14	HAMD_TOTAL	0.17	0.16
404	HAMD_15	HAMD_TOTAL	0.21	0.08
405	HAMD_16	HAMD_TOTAL	0.22	0.06
406	HAMD_17	HAMD_TOTAL	0.28	0.02

eTable 2. Multiple factors regression anaylsis of DC vaule and demographic and clinical factors

Cerebellum_r

term	estimate	std.error	statistic	p.value
(Intercept)	0.96	0.55	1.73	0.09
age	0.00	0.02	0.04	0.97
sex	-0.04	0.09	-0.51	0.61
duration	0.01	0.01	0.95	0.35
education	0.18	0.14	1.31	0.20
Clinical_features	0.02	0.03	0.74	0.46
HAMD_1	-0.01	0.05	-0.14	0.89
HAMD_2	-0.01	0.05	-0.15	0.88
HAMD_3	0.05	0.04	1.17	0.25
HAMD_4	0.07	0.04	1.57	0.12
HAMD_5	-0.04	0.06	-0.60	0.55
HAMD_6	0.07	0.05	1.48	0.15
HAMD_7	0.02	0.06	0.38	0.71
HAMD_8	0.03	0.06	0.45	0.66
HAMD_9	-0.12	0.05	-2.37	0.06
HAMD_10	0.05	0.04	1.19	0.24
HAMD_11	-0.01	0.04	-0.14	0.89
HAMD_12	0.01	0.05	0.29	0.77
HAMD_13	-0.07	0.06	-1.20	0.24
HAMD_14	0.01	0.05	0.25	0.80
HAMD_15	-0.04	0.05	-0.91	0.37
HAMD_16	-0.05	0.05	-0.97	0.34
HAMD_17	-0.06	0.12	-0.47	0.64

Lingual_l

term	estimate	std.error	statistic	p.value
(Intercept)	1.20	0.85	1.42	0.16
age	-0.01	0.03	-0.15	0.88
sex	-0.14	0.13	-1.10	0.28
duration	-0.04	0.02	-2.00	0.06
education	0.24	0.22	1.11	0.27
Clinical_features	-0.03	0.04	-0.80	0.43
HAMD_1	0.18	0.08	2.25	0.07
HAMD_2	-0.03	0.08	-0.32	0.75
HAMD_3	0.00	0.06	-0.05	0.96
HAMD_4	-0.03	0.07	-0.38	0.70
HAMD_5	-0.10	0.09	-1.06	0.29
HAMD_6	0.06	0.08	0.76	0.45
HAMD_7	-0.15	0.09	-1.78	0.08
HAMD_8	-0.23	0.09	-2.21	0.08

HAMD_9	0.03	0.08	0.35	0.72
HAMD_10	0.11	0.07	1.65	0.11
HAMD_11	-0.03	0.07	-0.45	0.65
HAMD_12	0.08	0.08	1.00	0.32
HAMD_13	0.04	0.09	0.47	0.64
HAMD_14	-0.06	0.08	-0.83	0.41
HAMD_15	-0.02	0.08	-0.24	0.81
HAMD_16	0.17	0.08	2.07	0.06
HAMD_17	0.16	0.19	0.85	0.40

Insula_r

term	estimate	std.error	statistic	p.value
(Intercept)	1.43	0.82	1.74	0.09
age	0.01	0.03	0.22	0.83
sex	-0.01	0.13	-0.10	0.92
duration	0.00	0.02	-0.08	0.94
education	-0.35	0.21	-1.69	0.10
Clinical_features	-0.02	0.04	-0.46	0.64
HAMD_1	-0.02	0.08	-0.23	0.82
HAMD_2	-0.07	0.08	-0.82	0.42
HAMD_3	-0.04	0.06	-0.60	0.55
HAMD_4	0.01	0.07	0.21	0.83
HAMD_5	-0.05	0.09	-0.54	0.59
HAMD_6	-0.05	0.07	-0.74	0.46
HAMD_7	0.00	0.08	0.02	0.98
HAMD_8	-0.08	0.09	-0.93	0.35
HAMD_9	0.00	0.07	-0.05	0.96
HAMD_10	-0.02	0.06	-0.29	0.77
HAMD_11	0.03	0.06	0.45	0.66
HAMD_12	0.01	0.07	0.14	0.89
HAMD_13	0.01	0.08	0.11	0.91
HAMD_14	-0.03	0.07	-0.35	0.72
HAMD_15	0.03	0.07	0.42	0.68
HAMD_16	-0.05	0.08	-0.58	0.56
HAMD_17	-0.14	0.18	-0.79	0.43

Insula_l

term	estimate	std.error	statistic	p.value
(Intercept)	1.24	0.80	1.54	0.13
age	0.00	0.03	-0.02	0.98
sex	-0.11	0.12	-0.90	0.37
duration	0.00	0.02	0.07	0.94
education	-0.20	0.20	-1.00	0.32
Clinical_features	-0.01	0.04	-0.31	0.75
HAMD_1	0.03	0.08	0.41	0.69
HAMD_2	0.05	0.08	0.61	0.55

HAMD_3	-0.01	0.06	-0.10	0.92
HAMD_4	0.04	0.07	0.56	0.58
HAMD_5	0.01	0.09	0.10	0.92
HAMD_6	-0.05	0.07	-0.73	0.47
HAMD_7	0.01	0.08	0.15	0.88
HAMD_8	-0.16	0.09	-1.84	0.07
HAMD_9	-0.07	0.07	-1.00	0.32
HAMD_10	-0.04	0.06	-0.62	0.54
HAMD_11	0.01	0.06	0.22	0.83
HAMD_12	-0.03	0.07	-0.41	0.68
HAMD_13	0.05	0.08	0.65	0.52
HAMD_14	-0.10	0.07	-1.36	0.18
HAMD_15	0.02	0.07	0.24	0.81
HAMD_16	-0.01	0.08	-0.13	0.90
HAMD_17	-0.19	0.18	-1.05	0.30

Superior_parietal_r

term	estimate	std.error	statistic	p.value
(Intercept)	1.71	0.92	1.85	0.07
age	-0.04	0.04	-1.01	0.32
sex	-0.04	0.14	-0.27	0.79
duration	-0.02	0.02	-0.83	0.41
education	-0.11	0.24	-0.46	0.65
Clinical_features	-0.01	0.04	-0.14	0.89
HAMD_1	0.04	0.09	0.41	0.68
HAMD_2	0.07	0.09	0.80	0.43
HAMD_3	0.05	0.07	0.73	0.47
HAMD_4	0.12	0.07	1.58	0.12
HAMD_5	0.03	0.10	0.28	0.78
HAMD_6	0.03	0.08	0.32	0.75
HAMD_7	-0.09	0.09	-0.95	0.35
HAMD_8	0.08	0.10	0.77	0.45
HAMD_9	-0.13	0.08	-1.61	0.11
HAMD_10	0.06	0.07	0.86	0.39
HAMD_11	0.00	0.07	-0.07	0.95
HAMD_12	0.01	0.08	0.16	0.87
HAMD_13	0.01	0.09	0.07	0.94
HAMD_14	-0.05	0.08	-0.59	0.56
HAMD_15	-0.08	0.08	-0.95	0.35
HAMD_16	-0.05	0.09	-0.62	0.54
HAMD_17	-0.27	0.20	-1.31	0.20

Superior_parietal_l

term	estimate	std.error	statistic	p.value
(Intercept)	0.79	0.98	0.81	0.42
age	-0.01	0.04	-0.19	0.85

sex	-0.17	0.15	-1.12	0.27
duration	0.01	0.02	0.64	0.53
education	0.01	0.25	0.04	0.97
Clinical_features	0.07	0.05	1.53	0.13
HAMD_1	-0.06	0.09	-0.68	0.50
HAMD_2	0.08	0.10	0.88	0.38
HAMD_3	0.08	0.07	1.12	0.27
HAMD_4	0.05	0.08	0.63	0.53
HAMD_5	0.00	0.11	0.02	0.98
HAMD_6	0.05	0.09	0.60	0.55
HAMD_7	0.03	0.10	0.27	0.79
HAMD_8	0.14	0.11	1.34	0.19
HAMD_9	-0.14	0.09	-1.56	0.13
HAMD_10	0.05	0.08	0.67	0.50
HAMD_11	0.06	0.08	0.75	0.46
HAMD_12	-0.03	0.09	-0.29	0.78
HAMD_13	0.06	0.10	0.61	0.54
HAMD_14	-0.08	0.09	-0.91	0.37
HAMD_15	-0.02	0.09	-0.22	0.83
HAMD_16	-0.05	0.09	-0.55	0.59
HAMD_17	0.00	0.22	0.01	0.99

Reference

[1]. Gao, Y., et al., Abnormal Degree Centrality as a Potential Imaging Biomarker for Right Temporal Lobe Epilepsy: A Resting-state Functional Magnetic Resonance Imaging Study and Support Vector Machine Analysis. Neuroscience, 2022. 487: p. 198-206.

[2]. Buckner RL, Sepulcre J, Talukdar T, Krienen FM, Liu H, Hedden T, Andrews-Hanna JR, Sperling RA, Johnson KA. Cortical hubs revealed by intrinsic functional connectivity: mapping, assessment of stability, and relation to Alzheimer's disease. J Neurosci. 2009 Feb 11;29(6):1860-73. doi: 10.1523/JNEUROSCI.5062-08.2009. PMID: 19211893; PMCID: PMC2750039.

[3]. Palaniyappan L, Liddle PF. Diagnostic discontinuity in psychosis: a combined study of cortical gyrification and functional connectivity. Schizophr Bull. 2014 May;40(3):675-84. doi: 10.1093/schbul/sbt050. Epub 2013 Apr 24. PMID: 23615812; PMCID: PMC3984507.

[4]. Takeuchi, H., et al., Degree centrality and fractional amplitude of low-frequency oscillations associated with Stroop interference. Neuroimage, 2015. 119: p. 197-209.

[5]. Gao, C., et al., Decreased Subcortical and Increased Cortical Degree Centrality in a Nonclinical College Student Sample with Subclinical Depressive Symptoms: A Resting-State fMRI Study. Front Hum Neurosci, 2016. 10: p. 617.

[6]. Shimizu Y, Yoshimoto J, Toki S, et al. Toward probabilistic di- agnosis and understanding of depression based on func- tional MRI data analysis with logistic group LASSO. PLoS One. 2015;10(5):e0123524.
